# Supplementary material for: Sponsorship of national and regional professional paediatrics associations by companies that make breast-milk substitutes: evidence from a review of official websites
Source: BMJ Open. 2019 Aug 10;9(8):e029035. doi: 10.1136/bmjopen-2019-029035 (PMC6701639; doi:10.1136/bmjopen-2019-029035)
Supplement: Author's manuscript [file bmjopen-2019-029035.draft_revisions.pdf]

BMJ Open is committed to open peer review. As part of this commitment we make the peer review history of every article we publish publicly available.

When an article is published we post the peer reviewers' comments and the authors' responses online. We also post the versions of the paper that were used during peer review. These are the versions that the peer review comments apply to.

The versions of the paper that follow are the versions that were submitted during the peer review process. They are not the versions of record or the final published versions. They should not be cited or distributed as the published version of this manuscript.

BMJ Open is an open access journal and the full, final, typeset and author-corrected version of record of the manuscript is available on our site with no access controls, subscription charges or pay-per-view fees (http://bmjopen.bmj.com).

If you have any questions on BMJ Open's open peer review process please email info.bmjopen@bmj.com

BMJ Open

Sponsorship of Professional Paediatrics Associations by Companies that Make Breast-milk Substitutes

Journal:	*BMJ Open*
Manuscript ID	bmjopen-2019-029035
Article Type:	Research
Date Submitted by the Author:	09-Jan-2019
Complete List of Authors:	Grummer-Strawn, Laurence; World Health Organization , Department of Nutrition for Health and Development<br>Holliday, Faire; Colorado School of Public Health<br>Jungo, Katharina; University of Bern, Institute of Primary Health Care<br>Rollins, Nigel; World Health Organization, Maternal, Newborn, Child and Adolescent Health
Keywords:	Conflict of interest, infant formula, pediatric associations, sponsorship, funding

SCHOLARONE™
Manuscripts

Sponsorship of Professional Paediatrics Associations by Companies that Make Breast-milk Substitutes

Laurence M Grummer-Strawn[1], Faire Holliday[2], Katharina Tabea Jungo[3] and Nigel Rollins[4]

Disclaimer: The authors alone are responsible for the views expressed in this article and they do not necessarily represent the decisions, policy or views of the institutions with which they are affiliated.

1. Department of Nutrition for Health and Development, World Health Organization, Geneva, Switzerland

2. Colorado School of Public Health, Fort Collins, Colorado, USA

3. Institute of Primary Health Care (BIHAM), University of Bern, Bern, Switzerland

4. Department of Maternal, Newborn, Child, and Adolescent Health, World Health Organization, Geneva, Switzerland

Correspondence to: Laurence M Grummer-Strawn (grummerstrawnl@who.int), Department of Nutrition for Health and Development, World Health Organization, Geneva, Switzerland

For peer review only

The Corresponding Author has the right to grant on behalf of all authors and does grant on behalf of all authors, a worldwide licence to the Publishers and its licensees in perpetuity, in all forms, formats and media (whether known now or created in the future), to i) publish, reproduce, distribute, display and store the Contribution, ii) translate the Contribution into other languages, create adaptations, reprints, include within collections and create summaries, extracts and/or, abstracts of the Contribution, iii) create any other derivative work(s) based on the Contribution, iv) to exploit all subsidiary rights in the Contribution, v) the inclusion of electronic links from the Contribution to third party material where-ever it may be located; and, vi) licence any third party to do any or all of the above.

The Corresponding Author has the right to grant on behalf of all authors and does grant on behalf of all authors, an exclusive licence on a worldwide basis to the BMJ Publishing Group Ltd to permit this article (if accepted) to be published in BMJ editions and any other BMJPGL products and sublicences such use and exploit all subsidiary rights, as set out in our licence

All authors have completed the Unified Competing Interest form and declare: no support from any organisation for the submitted work; no financial relationships with any organisations that might have an interest in the submitted work in the previous three years, no other relationships or activities that could appear to have influenced the submitted work.

The lead author affirms that the manuscript is an honest, accurate, and transparent account of the study being reported; that no important aspects of the study have been omitted; and that any discrepancies from the study as planned have been explained.

For peer review only

For peer review only - http://bmjopen.bmj.com/site/about/guidelines.xhtml

Contributors: LGS conceived the project, drafted the overall paper and tables and is responsible for the overall content as guarantor. FH oversaw and conducted the data collection and managed the data repository. KJ participated in the data collection, analysed the data, conducted the data analyses, and contributed part of the text. NR contributed to the project design and suggested significant revisions to the paper. All four authors reviewed the final revision.

Funding: All authors are staff or interns at the World Health Organization. No separate funding was obtained for this study.

Competing interests: None declared.

Patient consent: Not applicable.

Ethics approval: Not applicable since no human subjects were involved.

Data sharing statement: The data used to support the findings of this study are included within the article. No additional data are available.

For peer review only

For peer review only - http://bmjopen.bmj.com/site/about/guidelines.xhtml

Abstract

Objectives: Professional paediatrics associations have an important role to play in promoting the highest standard of care for women and children. Education and guidelines must be made in the best interests of patients. Given the importance of breastfeeding for the health, development and survival of infants, children and mothers, paediatric associations have a particular responsibility to avoid conflicts of interest with companies that manufacture breast-milk substitutes (BMS). The objective of this study was to investigate the extent to which national and regional paediatric associations are sponsored by BMS companies.

Methods: Data were collected on national paediatric associations based on online searches of websites and Facebook pages. Sites were examined for evidence of financial sponsorship by the breast-milk substitute industry, including funding of journals, newsletters, or other publications, conferences and events, scholarships, fellowship, grants, and awards. Payment for services, such as exhibitor space at conferences or events and paid advertisements in publications, was also noted.

Results: Overall, 60 of the 114 (53%) paediatric associations with a website or Facebook account documented sponsorship from breast-milk substitute companies. The most common type of sponsorship was of conferences or other events. The prevalence of conference sponsorship is highest in Europe and the Americas, where about half of the associations have BMS company-sponsored conferences. Thirty-one associations (27%) indicated that they received funding from BMS companies as payment for advertisements or exhibitor space. Only 18 associations (16%) have conflict of interest policies, guidelines, or criteria posted online.

Conclusion: Despite the well-documented importance of breastfeeding and the widespread recognition that commercial influences can shape the behaviours of health care professionals, national and regional paediatric associations commonly accept funding from companies that manufacture and distribute BMS. Paediatric Associations should function without the influence of commercial interests.

For peer review only - http://bmjopen.bmj.com/site/about/guidelines.xhtml

Strengths and limitations of this study

- This is the first study to systematically document the extent of conflicts of interest in national paediatric associations with regard to manufacturers of breast-milk substitutes.
- Data were available for 75% of national paediatric associations.
- Data were objectively collected and analysed based on online documentation.
- Funding that has not been documented on websites was not captured, potentially leading to an underestimate of the extent of industry funding.
- This study was not able to capture information on the amount of funding received or on how it compares to funding from other sources.

For peer review only

For peer review only - http://bmjopen.bmj.com/site/about/guidelines.xhtml

Sponsorship of Professional Paediatrics Associations by Companies that Make Breast-milk Substitutes

Laurence M. Grummer-Strawn, Faire Holliday, Katharina Tabea Jungo and Nigel Rollins

Introduction/background

Breastfeeding is critical for the health, development and survival of infants, children and mothers. Recent analyses have concluded that an estimated 820,000 infant and child deaths still occur each year from substandard breastfeeding practices or non-breastfeeding (Victora et al., 2016). Nearly half of diarrhoea episodes and one third of respiratory infections are due to inadequate breastfeeding practices. Longer breastfeeding is associated with a 13 percent reduction in the likelihood of overweight and obesity and a 35 percent reduction in the incidence of type 2 diabetes. An estimated 20 000 maternal deaths from breast cancer could be prevented each year by improving breastfeeding. Economically, increasing rates of breastfeeding could add US$ 300 billion to the global economy annually, by helping to foster smarter, more productive workers and leaders (Rollins et al., 2016).

Professional Medical Associations (PMAs) have an important role to play in promoting the highest standard of care to the general population (Schofferman et al., 2013; Fabbri et al., 2016). By investing in research, education, the creation of clinical guidelines, and advocacy, they help to shape their respective fields. These associations are often viewed as a preeminent source of knowledge on health behaviours. It is therefore imperative that they act independently and without the influence of commercial interests.

Given the importance of breastfeeding, health professional advice and support for breastfeeding should not be influenced by the commercial interests of companies that manufacture breast-milk substitutes (BMS). The dangers of involvement of BMS companies in health care have long been recognized. The

For peer review only - http://bmjopen.bmj.com/site/about/guidelines.xhtml

International Code of Marketing of Breast-milk Substitutes, adopted by the World Health Assembly in 1981, delineates a number of steps to limit the ways in which industry uses health care workers to promote its products (WHO, 1981). In 2005, the World Health Assembly urged countries to "ensure that financial support and other incentives for programmes and health professionals working in infant and young child health do not create conflicts of interest" (WHO, 2005).

The 69th World Health Assembly passed a resolution on ending inappropriate promotion of foods for infants and young children (WHO, 2016a). This document reconfirmed the World Health Organization's commitment to ending unethical marketing practices by private sector entities and directly called on health professionals to "fulfil their essential role in providing parents and other caregivers with information and support on optimal infant and young child feeding practices and to implement the guidance recommendations" (WHO, 2016b, paragraph 4). The guidance recommendations further state that health professional associations should not "accept equipment or services from companies that market foods for infants and young children; accept gifts or incentives from such companies…[or] allow such companies to sponsor meetings of health professionals and scientific meetings" (WHO, 2016b, paragraph 17).

Although anecdotal information exists about the relationship between the breast-milk substitute industry and paediatric associations, it has not yet been examined in a systematic way. The objective of this study was to investigate the extent to which national and regional paediatric associations are sponsored by breast-milk substitute companies.

Methods

For peer review only - http://bmjopen.bmj.com/site/about/guidelines.xhtml

Data were collected on national (n=146) or regional (n=6) paediatric associations listed on the webpage of the International Paediatric Association (IPA) (International Paediatric Association, 2017).

Online searches were conducted for evidence of funding of paediatric associations by the breast-milk substitute industry. This involved first determining which associations had official websites or, in the absence of official websites, Facebook accounts. Searches were then conducted within the online presence for these associations. For associations with a website, searches were conducted on the main website, as well as any associated journal websites, conference websites, and charity or non-profit branch or foundation websites, as available.

In addition, the Facebook accounts of ten randomly-selected associations that also had official websites were searched. As this process did not render any information in addition to that which was found on the website itself, no additional Facebook accounts were searched.

Data were collected by two research assistants in June-August 2017. Reliability was assessed by having both research assistants conduct searches for the same ten member associations and compare results. After this process, the remaining associations were divided and assigned to each research assistant. Some websites were in a language that was familiar to one research assistant or the other, and were therefore assigned accordingly. Websites in languages other than English, French, German, Italian, Portuguese or Spanish were translated using online translators and additional interpretation was sometimes provided by other WHO staff or interns. The rest were divided randomly.

Searches were also conducted on websites of breast-milk substitute companies. Due to the high number of such companies (and national affiliates), it was impossible to identify or search the websites of all. In order to be systematic, the websites of “infant formula” companies listed on the Baby Milk Action website were searched (Baby Milk Action, 2017).

For peer review only - http://bmjopen.bmj.com/site/about/guidelines.xhtml

Information was collected on the purpose of funding (Table 1) and classified as either “sponsorship” or “payment for services”. Funding was considered to be “sponsorship” if there appeared to be no specific services provided to the donor other than acknowledgement. Funding was considered to be “payment for services” if the paediatric association provided direct benefits to the company, such as through advertisements in a publication or exhibition space at a conference.

Data were entered into a spreadsheet and reviewed by both research assistants to double-check the relevance of the information. For each category of funding, “sponsorship” or “payment for services”, the association was counted as “yes” if there was evidence of financial contribution from BMS companies in that category. In addition to funding, information was collected on whether the association had policies or criteria on conflicts of interest available online.

For peer review only

Patient and public involvement

No patients were involved in the study.

Results

Of the 152 paediatric associations, websites were identified for 109 and an additional 5 had Facebook pages, yielding a total of 114 associations with an online presence (75%). Overall, 68 of the paediatric associations with an online presence (60%) documented receipt of some kind of financial support from breast-milk substitute companies, either in the form of sponsorship or payment for services (Table 2). This was highest in the Americas region, with 23 of 28 (82%) associations receiving some sort of funding from BMS companies. In Europe, 21 of 32 (66%) associations received BMS funding, while in Asia and

For peer review only - http://bmjopen.bmj.com/site/about/guidelines.xhtml

Africa it was 15 of 30 (50%) and 8 of 21 (38%), respectively. One association in Oceania was found to have received funding from breast-milk substitute companies. However, there are only three paediatric associations identified in this region, and the results should be considered accordingly.

Sponsorship was the most common type of funding received by paediatric associations. Overall, 60 of 114 (53%) websites of paediatric associations indicated sponsorship by BMS companies. Forty-three associations (38%) have conferences or other events sponsored by BMS companies. The prevalence of conference sponsorship is highest in Europe and the Americas, where about half of the associations have BMS company-sponsored conferences.

In addition to conferences, paediatric associations may receive other types of sponsorship from breast-milk substitute companies, though less frequently. Fifteen associations (13%) receive general sponsorship of the association or its website, 10 associations (9%) receive sponsorship for scholarships, awards, grants, or fellowships and 4 (4%) have publications that are sponsored.

Documentation of association sponsorship was also found on BMS company websites, although most of this information was captured on the association websites. While 16 associations were identified as recipients of funding on BMS company websites, only 3 of these associations did not already have documentation of this on their own website. In general, it was not possible to determine the purpose of the sponsorship on the BMS company websites.

Thirty-one associations (27%) indicated that they received funding from BMS companies as payment for services. This was highest in the Americas, with 14 of 28 (50%), and in Europe, with 10 of 21 (31%). Fourteen (12%) were found to have BMS company advertising in their online publications (journals, magazines, or newsletters). Worldwide, only one fifth (22 of 114) of the associations had exhibitions by the breast-milk substitute industry at conferences.

For peer review only - http://bmjopen.bmj.com/site/about/guidelines.xhtml

Only eighteen (16%) associations published conflict of interest policies, guidelines, or criteria. Associations that receive financial sponsorship from BMS companies are more likely to have a conflict of interest policy (13 of 60 associations, or 22%) than those that are not sponsored (5 of 54 associations, or 9%).

Discussion

Despite the well-documented importance of breastfeeding and the widespread recognition of how commercial influences shape the behaviours of health care professionals, national and regional paediatric associations commonly accept funding from companies that make BMS. This study found that 53 percent of association websites acknowledge receiving sponsorship from BMS companies. In addition, when payment for advertisements or exhibitor space is included, it can be seen that 60 percent of associations receive financial support from BMS companies.

A conflict of interest occurs when a set of conditions in which professional judgment concerning a primary interest (such as a patient's welfare or the validity of research) tends to be unduly influenced by a secondary interest (such as financial gain) (WHO, 2016c). Rodwin (2011) defined it as when an individual has an obligation to serve a party or perform a role and the individual has either incentives or conflicting loyalties which encourage the individual to act in ways that breach his/her obligations.

Each type of sponsorship presents its own challenges and has the potential to create a conflict of interest. Perhaps of greatest concern is the widespread sponsorship of conferences and other events at which paediatricians meet and disseminate research. The impact of the BMS industry at these events—either as direct sponsors, sponsors of symposia, or presenters of information—is not to be underestimated. For example, as Fabbri et al. (2016) noted, funding of conferences and satellite symposia may bias the scientific content presented at such events.

For peer review only - http://bmjopen.bmj.com/site/about/guidelines.xhtml

Furthermore, accepting any kind of support from the industry "creates a sense of obligation and loyalty to the company in question" (Costello et al., 2017, p. 597). Even receiving foods and beverages sponsored by industry at these events or receiving conference materials bearing company logos may cause physicians to feel a subconscious onus to reciprocate (Rothman et al., 2009). These factors have the potential to influence what physicians prescribe to their patients (Fabbri et al., 2016; Rothman et al., 2009). Exhibitions by the breast-milk substitute industry at conferences are likewise concerning, particularly as participants are often required to walk through the exhibit hall to access scientific events.

Industry sponsorship of medical journals is concerning in that it may shape the content that is presented to health professionals. Funding for scholarships and grants has the potential to impact what topics are researched, thus influencing the field for years to come (Dalsing, 2011).

This study was unable to document the actual amount of funding provided by the BMS companies, as this information is rarely posted on public websites. Professional medical associations are not required to share their financial records (Schofferman et al., 2013). By one recent estimate, professional medical associations receive 30 to 50 percent of their sponsorship from industry (Dalsing, 2011). Many associations are currently dependent on funding from BMS companies for operating expenses.

Although refusing sponsorship from the BMS industry may reduce paediatric associations' budgets, there are alternatives that could lessen the impact of the financial relationships. Schofferman et al. (2013) suggests that associations raise their dues, increase recruitment, or downsize some of their more expensive activities. It has been estimated that the American Academy of Pediatrics could raise its dues $50 to cover the cost of refusing BMS sponsorship (Sharfstein and Silver, 2017).

Only 18 associations (16%) had posted online some sort of policy to manage conflicts of interest. Interestingly, associations with a conflict of interest policy are actually more likely to accept sponsorship

For peer review only - http://bmjopen.bmj.com/site/about/guidelines.xhtml

from BMS companies than those that do not have such policies. This finding is consistent with previous research by Fabbri et al. (2016), showing that Italian professional medical associations with a conflict of interest policy were no less likely to have sponsorship from industry as those without. It may be the case that conflict of interest policies actually make it easier to accept funding or it may be that once a decision has been made to accept funding, the association sees the need to write down a policy to justify the acceptance and to govern how the funds will be used.

Limitations

As the research was limited to Associations' online presence, some information was likely missed. No information was available for the 38 Associations with neither a website or Facebook page, although these Associations were generally in small countries and may be quite small Associations. Review of websites not in English, French, German, Italian, Portuguese or Spanish may not have been entirely complete. Not all funding may be acknowledged on websites. It was outside of the scope of this project to investigate print versions of publications, which may be more likely to display advertisements than online versions of journals. As a result, the extent of industry funding is likely underestimated.

This study was not able to capture information on the amount of funding received or on how it compares to funding from other sources.

Conclusion

This study has documented that paediatric associations regularly receive funding from BMS companies, particularly through sponsorship of conferences and meetings, as well as publications, scholarships, fellowship, grants, and awards. Paediatric associations are tasked above all else with safeguarding the

For peer review only - http://bmjopen.bmj.com/site/about/guidelines.xhtml

health of infants, children and mothers and promoting the highest standard of care, including the protection, promotion and support of breastfeeding. WHO recommends this should be without influence from industry. Policies on conflicts of interest are relatively rare and do not appear to limit the decision to accept funds from the BMS industry. In accordance with the World Health Assembly Resolution 69.9, paediatric associations should refuse sponsorship from the BMS industry and identify alternative funding models especially with respect to the management and style of conferences.

For peer review only

For peer review only - http://bmjopen.bmj.com/site/about/guidelines.xhtml

Table 1. Type of funding from breast-milk substitute companies identified by online searches

Category	Criteria
Sponsorship	
Funding of journals, newsletters, or other publications	• Written indication of breast-milk substitute companies as sponsors of the journal, magazine, or newsletter
Funding for conferences and events	• Breast-milk substitute company logos on conference webpage<br>• Written indication of breast-milk substitute companies as sponsors<br>• Written indication of satellite symposia or other events sponsored by breast-milk substitute companies
Funding for scholarships, fellowship, grants, and awards	• Written indication of breast-milk substitute companies as sponsors of scholarships, awards, grants, or fellowship funding
Funding for websites or general use	• Breast-milk substitute company logos on home page or sponsors/partners page<br>• Written indication of breast-milk substitute companies as sponsors Written promotion of a particular breast-milk substitute company
Payment for services	
Paid advertisements in publications	• Advertisements of breast-milk substitute companies in online publications
Exhibitor space at conferences or events	• Written indication of breast-milk substitute companies as exhibitors<br>• Photos of breast-milk substitute exhibitions at conferences
Associations' conflict of interest policies, guidelines, or criteria	• Existence of any official document of the association that mentions conflicts of interest (includes official conflict of interest documents, codes of ethics, association statutes, etc.)

For peer review only

For peer review only - http://bmjopen.bmj.com/site/about/guidelines.xhtml

Table 2. Number of paediatric associations that receive financial support[*] from manufacturers of breast-milk substitutes based on website review, by type of support

	World (N=114)	Africa (N=21)	Americas (N=28)	Asia (N=30)	Europe (N=32)	Oceania (N=3)
Any financial support n, (%)	68 (60%)	8 (38%)	23 (82%)	15 (50%)	21 (66%)	1 (33%)
Sponsorship n, (%)	60 (53%)	8 (38%)	20 (71%)	13 (43%)	19 (59%)	0 (0%)
Funding of journals, newsletters, or other publications	4 (4%)	0 (0%)	1 (4%)	3 (10%)	0 (0%)	0 (0%)
Funding for conferences and events	43 (38%)	7 (33%)	13 (46%)	7 (23%)	16 (50%)	0 (0%)
Funding for scholarships, fellowship, grants, and awards	10 (9%)	0 (0%)	5 (18%)	3 (10%)	2 (6%)	0 (0%)
Funding for websites or general use	15 (13%)	2 (10%)	5 (18%)	3 (10%)	5 (16%)	0 (0%)
Purpose of funding not stated	16 (14%)	5 (24%)	7 (25%)	1 (3%)	3 (9%)	0 (0%)
Payment for services n, (%)	31 (27%)	1 (5%)	14 (50%)	5 (17%)	10 (31%)	1 (33%)
Paid advertisements in publications	14 (12%)	1 (5%)	5 (18%)	3 (10%)	5 (16%)	0 (0%)
Exhibitor space at conferences or events	22 (19%)	1 (5%)	10 (36%)	2 (7%)	8 (25%)	1(33%)

[*] Financial support includes sponsorship, for which no specific services are provided to the donor other than acknowledgement, and payment for services.

For peer review only - http://bmjopen.bmj.com/site/about/guidelines.xhtml

References:

1. Baby Milk Action: IBFAN UK. (2017). Infant formula: companies and brands. Retrieved from http://archive.babymilkaction.org/pages/milkcom.html

2. Costello, A., Branca, F., Rollins, N., Stahlhofer, M., Grummer-Strawn L. (2017). Health professional associations and industry funding. The Lancet, 389, 597-598. Retrieved from http://ac.els-cdn.com/S0140673617302775/1-s2.0-S0140673617302775-main.pdf?_tid=93dfdd3e-7076-11e7-a26a-00000aab0f26&acdnat=1500904204_1c57f80892d4e227e118453de636cc13

3. Council of Medical Specialty Societies. (2015). Code for Interactions with Companies. Retrieved from https://cmss.org/wp-content/uploads/2016/02/CMSS-Code-for-Interactions-with Companies-Approved-Revised-Version-4.13.15-with-Annotations.pdf

4. Dalsing, M. (2011). Industry working with physicians through professional medical associations. *Journal of Vascular Surgery, 54 (18S)*, 41S-46S. Retrieved from http://www.sciencedirect.com/science/article/pii/S0741521411013711?via%3Dihub

5. Fabbri, A., Gregoraci, G., Tedesco, D., Ferretti, F., Gilardi, F., Iemmi, D., Lisi, C., Lorusso, A., Natali, F., Shahi, E. & Rinaldi, A. (2016). Conflict of interest between professional medical societies and industry: a crosssectional study of Italian medical societies' websites. *BMJ Open, 6,* 1-8. Retrieved from http://bmjopen.bmj.com/content/bmjopen/6/6/e011124.full.pdf

6. International Paediatric Association. (2017). Member Societies. Retrieved from http://ipa-world.org/society.php

7. Nissen, S. (2017). Conflicts of Interest and Professional Medical Associations: Progress and Remaining Challenges. *Journal of the American Medical Association, 317 (17)*, 1737-1738. Retrieved from http://jamanetwork.com.ezproxy2.library.colostate.edu/journals/jama/fullarticle/2623622

8. Rollins N.C., Bhandari N., Hajeebhoy N., Horton S., Lutter C.K., Martines J.C. et al (2106). Why invest, and what it will take to improve breastfeeding practices? *Lancet,* 387:491–504. doi:10.1016/S0140-6736(15)01044-2.

9. Rodwin, M.A., *Conflicts of Interest and the Future of Medicine: The United States, France and Japan*, Oxford: Oxford University Press, 2011. Pp. ix-375. ISBN 978 0 19 975548 6.

10. Rothman, D., McDonald, W., Berkowitz, C., Chimonas, S., DeAgelis, C., Hale, R., Nissen, S., Osborn, J., Scully J., J., Thomson, G. & Wofsy, D. (2009). Professional Medical Associations and Their Relationships With Industry: A Proposal for Controlling Conflict of Interest. *Journal of American Medical Association, 301(13), 1367-1372.* Retrieved from http://jamanetwork.com.ezproxy2.library.colostate.edu/journals/jama/fullarticle/183670

For peer review only - http://bmjopen.bmj.com/site/about/guidelines.xhtml

11. Schofferman, J., Eskay-Auerbach, M., Sawyer, L., Herring, S., Arnold, P & Muehlbauer, E. (2013). Conflict of interest and professional medical associations: the North American Spine Society experience. *The Spine Journal, 13,* 974-979. Retrieved from http://www.sciencedirect.com.ezproxy2.library.colostate.edu/science/article/pii/S1529943012005530?via%3Dihub

12. Sharfstein J.M. & Silver D.L. Relationships between the American Academy of Pediatrics and Infant Formula Companies. JAMA Pediatrics, 2017, E1-E2. Retrieved from http://jamanetwork.com/pdfaccess.ashx?url=/data/journals/peds/0/.

13. Siegel, M., Stedman, A., & Smith, K. (2015). Paediatric Professional Medical Associations and Industry Guideline Compliance. *Paediatrics, 136 (3)*, 528-533. Retrieved from http://paediatrics.aappublications.org/content/paediatrics/136/3/528.full.pdf

14. Thousand Days, UNICEF, & WHO. (2017). Investment Case for Breastfeeding. Retrieved from https://thousanddays.org/wp-content/uploads/Global-Breastfeeding-Collective_Case-For-Investment.pdf

15. Victora, C.G., Bahl, R., Barros, A.J., França, G.V., et al. Breastfeeding in the 21st century: epidemiology, mechanisms, and lifelong effect. *Lancet*, 2016; 387(10017), 475-490. https://doi.org/10.1016/S0140-6736(15)01024-7

16. World Health Organization (WHO). (1981). *International Code of Marketing of Breast-milk Substitutes.* Retrieved from http://www.who.int/nutrition/publications/code_english.pdf

17. World Health Organization, Forty-Ninth World Health Assembly. (1996). Resolution WHA 49.15 Infant and young child nutrition. Retrived from http://www.who.int/nutrition/topics/WHA49.15_iycn_en.pdf?ua=1

18. World Health Organization, Fifty-Eighth World Health Assembly. (2005). Resolution WHA58.32 Infant and young child nutrition. Retrieved from http://www.who.int/nutrition/topics/WHA58.32_iycn_en.pdf?ua=1

19. World Health Organization, Sixty-Ninth World Health Assembly. (2016a). Resolution WHA69.9 Ending inappropriate promotion of foods for infants and young children. Retrieved from http://apps.who.int/gb/ebwha/pdf_files/WHA69/A69_R9-en.pdf?ua=1&ua=1

20. World Health Organization, Sixty-Ninth World Health Assembly. (2016b). Provisional agenda item 12.1 Guidance on ending the inappropriate promotion of foods for infants and young children. Retrieved from http://apps.who.int/gb/ebwha/pdf_files/WHA69/A69_7Add1-en.pdf?ua=1

21. World Health Organization (WHO). (2016c). WHO Framework of Engagement with Non-State Actors (FENSA). Retrieved from http://www.who.int/about/collaborations/non-state-actors/en/

For peer review only - http://bmjopen.bmj.com/site/about/guidelines.xhtml

STROBE Statement—Checklist of items that should be included in reports of ***cross-sectional studies***

	Item No	Recommendation	Page #
**Title and abstract**	1	(*a*) Indicate the study's design with a commonly used term in the title or the abstract	3
		(*b*) Provide in the abstract an informative and balanced summary of what was done and what was found	3
**Introduction**			
Background/rationale	2	Explain the scientific background and rationale for the investigation being reported	4-5
Objectives	3	State specific objectives, including any prespecified hypotheses	5
**Methods**			
Study design	4	Present key elements of study design early in the paper	6-7
Setting	5	Describe the setting, locations, and relevant dates, including periods of recruitment, exposure, follow-up, and data collection	6
Participants	6	(*a*) Give the eligibility criteria, and the sources and methods of selection of participants	6
Variables	7	Clearly define all outcomes, exposures, predictors, potential confounders, and effect modifiers. Give diagnostic criteria, if applicable	7
Data sources/ measurement	8*	For each variable of interest, give sources of data and details of methods of assessment (measurement). Describe comparability of assessment methods if there is more than one group	7
Bias	9	Describe any efforts to address potential sources of bias	6
Study size	10	Explain how the study size was arrived at	6
Quantitative variables	11	Explain how quantitative variables were handled in the analyses. If applicable, describe which groupings were chosen and why	7
Statistical methods	12	(*a*) Describe all statistical methods, including those used to control for confounding	7
		(*b*) Describe any methods used to examine subgroups and interactions	7
		(*c*) Explain how missing data were addressed	7
		(*d*) If applicable, describe analytical methods taking account of sampling strategy	7
		(*e*) Describe any sensitivity analyses	N/A
**Results**			
Participants	13*	(a) Report numbers of individuals at each stage of study—eg numbers potentially eligible, examined for eligibility, confirmed eligible, included in the study, completing follow-up, and analysed	7
		(b) Give reasons for non-participation at each stage	N/A
		(c) Consider use of a flow diagram	N/A
Descriptive data	14*	(a) Give characteristics of study participants (eg demographic, clinical, social) and information on exposures and potential confounders	7
		(b) Indicate number of participants with missing data for each variable of interest	7
Outcome data	15*	Report numbers of outcome events or summary measures	7
Main results	16	(*a*) Give unadjusted estimates and, if applicable, confounder-adjusted estimates and their precision (eg, 95% confidence interval). Make clear	7-8

For peer review only - http://bmjopen.bmj.com/site/about/guidelines.xhtml

		which confounders were adjusted for and why they were included	
		(*b*) Report category boundaries when continuous variables were categorized	N/A
		(*c*) If relevant, consider translating estimates of relative risk into absolute risk for a meaningful time period	N/A
Other analyses	17	Report other analyses done—eg analyses of subgroups and interactions, and sensitivity analyses	N/A
**Discussion**			
Key results	18	Summarise key results with reference to study objectives	9
Limitations	19	Discuss limitations of the study, taking into account sources of potential bias or imprecision. Discuss both direction and magnitude of any potential bias	11
Interpretation	20	Give a cautious overall interpretation of results considering objectives, limitations, multiplicity of analyses, results from similar studies, and other relevant evidence	9-12
Generalisability	21	Discuss the generalisability (external validity) of the study results	11
**Other information**			
Funding	22	Give the source of funding and the role of the funders for the present study and, if applicable, for the original study on which the present article is based	2

*Give information separately for exposed and unexposed groups.

Note: An Explanation and Elaboration article discusses each checklist item and gives methodological background and published examples of transparent reporting. The STROBE checklist is best used in conjunction with this article (freely available on the Web sites of PLoS Medicine at http://www.plosmedicine.org/, Annals of Internal Medicine at http://www.annals.org/, and Epidemiology at http://www.epidem.com/). Information on the STROBE Initiative is available at www.strobe-statement.org.

For peer review only

For peer review only - http://bmjopen.bmj.com/site/about/guidelines.xhtml

BMJ Open

Sponsorship of National and Regional Professional Paediatrics Associations by Companies that Make Breast-milk Substitutes: Evidence from a Review of Official Websites

Journal:	*BMJ Open*
Manuscript ID	bmjopen-2019-029035.R1
Article Type:	Research
Date Submitted by the Author:	04-Apr-2019
Complete List of Authors:	Grummer-Strawn, Laurence; World Health Organization, Department of Nutrition for Health and Development<br>Holliday, Faire; Colorado School of Public Health<br>Jungo, Katharina; University of Bern, Institute of Primary Health Care<br>Rollins, Nigel; World Health Organization, Department of Maternal, Newborn, Child and Adolescent Health
**Primary Subject Heading**:	Paediatrics
Secondary Subject Heading:	Nutrition and metabolism
Keywords:	Conflict of interest, infant formula, pediatric associations, sponsorship, funding

SCHOLARONE™
Manuscripts

Sponsorship of National and Regional Professional Paediatrics Associations by Companies that Make Breast-milk Substitutes: Evidence from a Review of Official Websites

Laurence M Grummer-Strawn[1], Faire Holliday[2], Katharina Tabea Jungo[3] and Nigel Rollins[4]

For peer review only

Disclaimer: The authors alone are responsible for the views expressed in this article and they do not necessarily represent the decisions, policy or views of the institutions with which they are affiliated.

1. Department of Nutrition for Health and Development, World Health Organization, Geneva, Switzerland

2. Colorado School of Public Health, Fort Collins, Colorado, USA

3. Institute of Primary Health Care (BIHAM), University of Bern, Bern, Switzerland

4. Department of Maternal, Newborn, Child, and Adolescent Health, World Health Organization, Geneva, Switzerland

Correspondence to: Laurence M Grummer-Strawn (grummerstrawnl@who.int), Department of Nutrition for Health and Development, World Health Organization, Geneva, Switzerland

The Corresponding Author has the right to grant on behalf of all authors and does grant on behalf of all authors, a worldwide licence to the Publishers and its licensees in perpetuity, in all forms, formats and media (whether known now or created in the future), to i) publish, reproduce, distribute, display and store the Contribution, ii) translate the Contribution into other languages, create adaptations, reprints, include within collections and create summaries, extracts and/or, abstracts of the Contribution, iii) create any other derivative work(s) based on the Contribution, iv) to exploit all subsidiary rights in the Contribution, v) the inclusion of electronic links from the Contribution to third party material where-ever it may be located; and, vi) licence any third party to do any or all of the above.

The Corresponding Author has the right to grant on behalf of all authors and does grant on behalf of all authors, an exclusive licence on a worldwide basis to the BMJ Publishing Group Ltd to permit this article (if accepted) to be published in BMJ editions and any other BMJPGL products and sublicences such use and exploit all subsidiary rights, as set out in our licence

All authors have completed the Unified Competing Interest form and declare: no support from any organisation for the submitted work; no financial relationships with any organisations that might have an interest in the submitted work in the previous three years, no other relationships or activities that could appear to have influenced the submitted work.

The lead author affirms that the manuscript is an honest, accurate, and transparent account of the study being reported; that no important aspects of the study have been omitted; and that any discrepancies from the study as planned have been explained.

For peer review only

Contributors: LGS conceived the project, drafted the overall paper and tables and is responsible for the overall content as guarantor. FH oversaw and conducted the data collection and managed the data repository. KJ participated in the data collection, analysed the data, conducted the data analyses, and contributed part of the text. NR contributed to the project design and suggested significant revisions to the paper. All four authors reviewed the final revision.

Funding: All authors are staff or interns at the World Health Organization. No separate funding was obtained for this study.

Competing interests: None declared.

Patient consent: Not applicable.

Ethics approval: Not applicable since no human subjects were involved.

Data sharing statement: The data used to support the findings of this study are included within the article. No additional data are available.

For peer review only

Abstract

Objectives: Professional paediatrics associations have an important role to play in promoting the highest standard of care for women and children. Education and guidelines must be made in the best interests of patients. Given the importance of breastfeeding for the health, development and survival of infants, children and mothers, paediatric associations have a particular responsibility to avoid conflicts of interest with companies that manufacture breast-milk substitutes (BMS). The objective of this study was to investigate the extent to which national and regional paediatric associations are sponsored by BMS companies.

Methods: Data were collected on national paediatric associations based on online searches of websites and Facebook pages. Sites were examined for evidence of financial sponsorship by the breast-milk substitute industry, including funding of journals, newsletters, or other publications, conferences and events, scholarships, fellowship, grants, and awards. Payment for services, such as exhibitor space at conferences or events and paid advertisements in publications, was also noted.

Results: Overall, 68 (60%) of the 114 paediatric associations with a website or Facebook account documented receiving financial support from breast-milk substitute companies. Sponsorship, particularly of conferences or other events, was the most common type of financial support. The prevalence of conference sponsorship is highest in Europe and the Americas, where about half of the associations have BMS company-sponsored conferences. Thirty-one associations (27%) indicated that they received funding from BMS companies as payment for advertisements or exhibitor space. Only 18 associations (16%) have conflict of interest policies, guidelines, or criteria posted online.

Conclusion: Despite the well-documented importance of breastfeeding and the widespread recognition that commercial influences can shape the behaviours of health care professionals, national and regional paediatric associations commonly accept funding from companies that manufacture and distribute BMS. Paediatric Associations should function without the influence of commercial interests.

For peer review only

Strengths and limitations of this study

- This is the first study to systematically document the extent of conflicts of interest in national paediatric associations with regard to manufacturers of breast-milk substitutes.
- Data from websites were available for 75% of national paediatric associations.
- Data were objectively collected and analysed based on online documentation.
- Funding that has not been documented on websites was not captured, potentially leading to an underestimate of the extent of industry funding.
- This study was not able to capture information on the amount of funding received or on how it compares to funding from other sources.

For peer review only

Evidence of Sponsorship by Companies that Make Breast-milk Substitutes on the Websites of National and Regional Paediatric Associations

Laurence M. Grummer-Strawn, Faire Holliday, Katharina Tabea Jungo and Nigel Rollins

Introduction/background

Breastfeeding is critical for the health, development and survival of infants, children and mothers. Recent analyses have concluded that an estimated 820,000 infant and child deaths still occur each year from substandard breastfeeding practices or non-breastfeeding.[1] Nearly half of diarrhoea episodes and one third of respiratory infections are due to inadequate breastfeeding practices. Longer breastfeeding is associated with a 13 percent reduction in the likelihood of overweight and obesity and a 35 percent reduction in the incidence of type 2 diabetes. An estimated 20 000 maternal deaths from breast cancer could be prevented each year by improving breastfeeding. Economically, increasing rates of breastfeeding could add US$ 300 billion to the global economy annually, by helping to foster smarter, more productive workers and leaders.[2]

Marketing of breast-milk substitutes (BMS) has effectively reduced rates of breastfeeding. Globally, sales of breast-milk substitutes are growing almost eight times as quickly as the world's population.[3] It is estimated that the six largest BMS manufacturers spend over $7 billion annually on marketing activities.[4] A significant portion of this marketing is targeted at health care providers and institutions. Given the importance of breastfeeding, health professional advice and support for breastfeeding should not be influenced by the commercial interests of BMS manufacturers.

The widespread involvement of BMS companies in health care has long been recognized and documented.[5-7] Recent studies in Mexico, Chile, and Ecuador have reported extensive use of BMS

For peer review only

promotional materials in health facilities, distribution of free formula samples, gifts to health workers, donations of equipment, and sponsorship of events.[8-10] In Bangladesh, many mothers are advised by health care workers, esp. in private clinics, to use BMS without any prior counselling on breastfeeding.[4] Health workers in China were paid by a BMS company to recommend infant formula to new or expectant mothers, leading to a $12million settlement against the company.[11]

The International Code of Marketing of Breast-milk Substitutes, adopted by the World Health Assembly in 1981, delineates a number of steps to limit the ways in which industry uses health care workers to promote its products.[12] In 2005, the World Health Assembly urged countries to "ensure that financial support and other incentives for programmes and health professionals working in infant and young child health do not create conflicts of interest".[13] The 69th World Health Assembly (2016) passed a resolution on WHO's "Guidance on ending inappropriate promotion of foods for infants and young children", calling on health professionals to "fulfil their essential role in providing parents and other caregivers with information and support on optimal infant and young child feeding practices and to implement the guidance recommendations" (paragraph 4).[14] The guidance recommendations[15] state that health professional associations should not "accept equipment or services from companies that market foods for infants and young children; accept gifts or incentives from such companies…[or] allow such companies to sponsor meetings of health professionals and scientific meetings" (paragraph 17). (The Implementation Manual[16] for the Guidance provides more information).

Although anecdotal information exists about the relationship between the breast-milk substitute industry and paediatric associations, it has not yet been examined in a systematic way. The objective of this study was to investigate the extent to which national and regional paediatric associations are sponsored by breast-milk substitute companies.

For peer review only

Methods

Data were collected on national (n=146) or regional (n=6) paediatric associations listed on the webpage of the International Paediatric Association (IPA).[17]

Online searches were conducted for evidence of funding of paediatric associations by the breast-milk substitute industry. This involved first determining which associations had official websites or, in the absence of official websites, Facebook accounts. Searches were then conducted within the online presence for these associations. For associations with a website, searches were conducted on the main website, as well as any associated journal websites, conference websites, and charity or non-profit branch or foundation websites, as available.

In addition, the Facebook accounts of ten randomly-selected associations that also had official websites were searched. As this process did not render any information in addition to that which was found on the website itself, no additional Facebook accounts were searched.

Data were collected by two research assistants in June-August 2017. Reliability was assessed by having both research assistants conduct searches for the same ten member associations and compare results. Minor discrepancies were encountered as to where on the website certain information was encountered, but in no case was the overall assessment of receipt of funding from BMS companies different. After this process, the remaining associations were divided and assigned to each research assistant. Some websites were in a language that was familiar to one research assistant or the other and were therefore assigned accordingly. Websites in languages other than English, French, German, Italian, Portuguese or Spanish were translated using online translators and additional interpretation was sometimes provided by other WHO staff or interns. The rest were divided randomly.

For peer review only

The websites were examined for any logos or names of BMS companies found in acknowledgments, funding sources, advertisements, and lists of exhibitors or sponsors of conferences. Where funders or sponsors were unknown, additional web searches were conducted to fully understand the nature of the donor. Home pages and all sub-pages, PowerPoint presentations, event photos, and online documents such as pamphlets, education sheets, newsletters, and publicly accessible journals were included in the search. Content that required a membership to access, such as subscription journals, were not reviewed.

Searches were also conducted on websites of breast-milk substitute companies. Due to the high number of such companies (and national affiliates), it was impossible to identify or search the websites of all. In order to be systematic, the websites of "infant formula" companies listed on the Baby Milk Action website were searched.[18]

Information was collected on the purpose of funding (Table 1) and classified as either "sponsorship" or "payment for services". Funding was considered to be "sponsorship" if there appeared to be no specific services provided to the donor other than acknowledgement. Funding was considered to be "payment for services" if the paediatric association provided direct benefits to the company, such as through advertisements in a publication or exhibition space at a conference.

Data were entered into a spreadsheet and checked by both research assistants to verify the relevance of the information. For each category of funding, "sponsorship" or "payment for services", the association was counted as "yes" if there was evidence of financial contribution from BMS companies in that category. In addition to funding, information was collected on whether the association had policies or criteria on conflicts of interest available online.

For peer review only

Patient and public involvement

No patients were involved in the study.

Results

Of the 152 paediatric associations, websites were identified for 109 and an additional 5 had Facebook pages, yielding a total of 114 associations with an online presence (75%). Overall, 68 of the paediatric associations with an online presence (60%) documented receipt of some kind of financial support from breast-milk substitute companies, either in the form of sponsorship or payment for services (Table 2). This was highest in the Americas region, with 23 of 28 (82%) associations receiving some sort of funding from BMS companies. In Europe, 21 of 32 (66%) associations received BMS funding, while in Asia and Africa it was 15 of 30 (50%) and 8 of 21 (38%), respectively. One association in Oceania was found to have received funding from breast-milk substitute companies. However, there are only three paediatric associations identified in this region, and the results should be considered accordingly.

Sponsorship was the most common type of funding received by paediatric associations. Overall, 60 of 114 (53%) websites of paediatric associations indicated sponsorship by BMS companies. Forty-three associations (38%) have conferences or other events sponsored by BMS companies. The prevalence of conference sponsorship is highest in Europe and the Americas, where about half of the associations have BMS company-sponsored conferences.

In addition to conferences, paediatric associations may receive other types of sponsorship from breast-milk substitute companies, though less frequently. Fifteen associations (13%) receive general

For peer review only

sponsorship of the association or its website, 10 associations (9%) receive sponsorship for scholarships, awards, grants, or fellowships and 4 (4%) have publications that are sponsored.

Thirty-one associations (27%) indicated that they received funding from BMS companies as payment for services (e.g. journal advertisements or exhibition space at conferences). This was highest in the Americas, with 14 of 28 (50%), and in Europe, with 10 of 21 (31%). Fourteen (12%) were found to have BMS company advertising in their online publications (journals, magazines, or newsletters). Worldwide, only one fifth (22 of 114) of the associations had exhibitions by the breast-milk substitute industry at conferences.

Documentation of association sponsorship was also found on BMS company websites, although most of this information was captured on the association websites. While 16 associations were identified as recipients of funding on BMS company websites, only 3 of these associations did not already have documentation of this on their own website. In general, it was not possible to determine the purpose of the sponsorship on the BMS company websites.

Only eighteen (16%) associations published conflict of interest policies, guidelines, or criteria. Many of these address conflicts of interest among individuals in leadership positions or the need to declare interests when making presentations. Policies on sponsorship or funding typically gave general criteria that donors cannot compromise the vision, mission, or values of the association but did not specify how this would be determined. Two associations listed specific industries that they would not work with (e.g. tobacco and arms), but in neither case were breast-milk substitute manufacturers on that list.

Associations that receive financial sponsorship from BMS companies are more likely to have a conflict of interest policy (13 of 60 associations, or 22%) than those that are not sponsored (5 of 54 associations, or 9%).

For peer review only

Discussion

Despite the well-documented importance of breastfeeding and the widespread recognition of how commercial influences shape the behaviours of health care professionals, national and regional paediatric associations commonly accept funding from companies that make BMS. This study found that 53 percent of association websites acknowledge receiving sponsorship from BMS companies. In addition, when payment for advertisements or exhibitor space is included, it can be seen that 60 percent of associations receive financial support from BMS companies.

A conflict of interest occurs when a set of conditions in which professional judgment concerning a primary interest (such as a patient's welfare or the validity of research) tends to be unduly influenced by a secondary interest (such as financial gain).[19] Rodwin[20] defined it as when an individual has an obligation to serve a party or perform a role and the individual has either incentives or conflicting loyalties which encourage the individual to act in ways that breach his/her obligations.

Each type of sponsorship presents its own challenges and has the potential to create a conflict of interest. Perhaps of greatest concern is the widespread sponsorship of conferences and other events at which paediatricians meet and disseminate research. The impact of the BMS industry at these events—either as direct sponsors, sponsors of symposia, or presenters of information—is not to be underestimated. For example, as Fabbri et al.[21] noted, funding of conferences and satellite symposia may bias the scientific content presented at such events.

Furthermore, accepting any kind of support from the industry "creates a sense of obligation and loyalty to the company in question".[22] Even receiving foods and beverages sponsored by industry at these events or receiving conference materials bearing company logos may cause physicians to feel a

For peer review only

subconscious onus to reciprocate.[23] These factors have the potential to influence what physicians prescribe to their patients.[21,23] Exhibitions by the breast-milk substitute industry at conferences are likewise concerning, particularly as participants are often required to walk through the exhibit hall to access scientific events.

Industry sponsorship of medical journals is concerning in that it may shape the content that is presented to health professionals. Funding for scholarships and grants has the potential to impact what topics are researched, thus influencing the field for years to come.[24] Recommendations on clinical practice may be unduly influenced by close relationships between professional expert bodies and the BMS industry. For example, van Tulleken[25] has noted that prescriptions of specialist formula milks for cow's milk protein allergy have increased dramatically over the past decade based on the guidelines of several expert groups. Ten of the 12 authors of the 2012 European Society for Paediatric Gastroenterology, Hepatology, and Nutrition (ESPGHAN) guidelines on diagnosis and management of cow's-milk protein allergy[26] and all authors of the international Milk Allergy in Primary Care (iMAP) guideline on cow's milk allergy[27] declared financial interests with infant formula manufacturers.

This study was unable to document the actual amount of funding provided by the BMS companies, as this information is rarely posted on public websites. Professional medical associations are not required to share their financial records.[28] Dalsing[24] has estimated that professional medical associations receive 30 to 50 percent of their budgets from industry relationships. Many associations are currently dependent on funding from BMS companies for operating expenses.

Although refusing sponsorship from the BMS industry may reduce paediatric associations' budgets, there are alternatives that could lessen the impact of the financial relationships. Schofferman et al.[28] suggests that associations raise their dues, increase recruitment, or downsize some of their more

For peer review only

expensive activities. It has been estimated that the American Academy of Pediatrics could raise its dues $50 to cover the cost of refusing BMS sponsorship.[29]

Only 18 associations (16%) had posted online some sort of policy to manage conflicts of interest. Interestingly, associations with a conflict of interest policy are actually more likely to accept sponsorship from BMS companies than those that do not have such policies. This finding is consistent with previous research by Fabbri et al.,[21] showing that Italian professional medical associations with a conflict of interest policy were no less likely to have sponsorship from industry as those without. It may be the case that conflict of interest policies actually make it easier to accept funding or it may be that once a decision has been made to accept funding, the association sees the need to write down a policy to justify the acceptance and to govern how the funds will be used.

Strengths

This is the first time that the sponsorship of national and regional paediatric associations by breast-milk substitute companies has been documented in a systematic way. Out of 152 known associations, we found online information about 114 of them, allowing for a regional breakdown of patterns. Data were objectively collected and analysed. We were able to document the purpose of the funding received and examine the existence and content of policies about funding and conflicts of interest.

Limitations

As the research was limited to Associations' online presence, some information was likely missed. The associations were not contacted directly to confirm the completeness of the website documentation. No information was available for the 38 Associations with neither a website or Facebook page, although these Associations were generally in small countries and may be quite small Associations. Review of websites not in English, French, German, Italian, Portuguese or Spanish may not have been entirely

For peer review only

complete. Not all funding may be acknowledged on websites. It was outside of the scope of this project to investigate print versions of publications, which may be more likely to display advertisements than online versions of journals. As a result, the extent of industry funding is likely underestimated.

This study was not able to capture information on the amount of funding received or on how it compares to funding from other sources.

For peer review only

Conclusion

This study has documented that paediatric associations regularly receive funding from BMS companies, particularly through sponsorship of conferences and meetings, as well as publications, scholarships, fellowship, grants, and awards. Paediatric associations are tasked above all else with safeguarding the health of infants, children and mothers and promoting the highest standard of care, including the protection, promotion and support of breastfeeding. WHO recommends this should be without influence from industry. Policies on conflicts of interest are relatively rare and do not appear to limit the decision to accept funds from the BMS industry. In accordance with the World Health Assembly Resolution 69.9, paediatric associations should refuse sponsorship from the BMS industry and identify alternative funding models especially with respect to the management and style of conferences.

Table 1. Type of funding from breast-milk substitute companies identified by online searches

Category	Criteria
Sponsorship	
Funding of journals, newsletters, or other publications	• Written indication of breast-milk substitute companies as sponsors of the journal, magazine, or newsletter
Funding for conferences and events	• Breast-milk substitute company logos on conference webpage<br>• Written indication of breast-milk substitute companies as sponsors<br>• Written indication of satellite symposia or other events sponsored by breast-milk substitute companies
Funding for scholarships, fellowship, grants, and awards	• Written indication of breast-milk substitute companies as sponsors of scholarships, awards, grants, or fellowship funding
Funding for websites or general use	• Breast-milk substitute company logos on home page or sponsors/partners page<br>• Written indication of breast-milk substitute companies as sponsors Written promotion of a particular breast-milk substitute company
Payment for services	
Paid advertisements in publications	• Advertisements of breast-milk substitute companies in online publications
Exhibitor space at conferences or events	• Written indication of breast-milk substitute companies as exhibitors<br>• Photos of breast-milk substitute exhibitions at conferences
Associations' conflict of interest policies, guidelines, or criteria	• Existence of any official document of the association that mentions conflicts of interest (includes official conflict of interest documents, codes of ethics, association statutes, etc.)

For peer review only

Table 2. Number of paediatric associations that receive financial support* from manufacturers of breast-milk substitutes based on website review, by type of support

	World (N=114)	Africa (N=21)	Americas (N=28)	Asia (N=30)	Europe (N=32)	Oceania (N=3)
Any financial support n, (%)	68 (60%)	8 (38%)	23 (82%)	15 (50%)	21 (66%)	1 (33%)
Sponsorship n, (%)	60 (53%)	8 (38%)	20 (71%)	13 (43%)	19 (59%)	0 (0%)
Funding of journals, newsletters, or other publications	4 (4%)	0 (0%)	1 (4%)	3 (10%)	0 (0%)	0 (0%)
Funding for conferences and events	43 (38%)	7 (33%)	13 (46%)	7 (23%)	16 (50%)	0 (0%)
Funding for scholarships, fellowship, grants, and awards	10 (9%)	0 (0%)	5 (18%)	3 (10%)	2 (6%)	0 (0%)
Funding for websites or general use	15 (13%)	2 (10%)	5 (18%)	3 (10%)	5 (16%)	0 (0%)
Purpose of funding not stated	16 (14%)	5 (24%)	7 (25%)	1 (3%)	3 (9%)	0 (0%)
Payment for services n, (%)	31 (27%)	1 (5%)	14 (50%)	5 (17%)	10 (31%)	1 (33%)
Paid advertisements in publications	14 (12%)	1 (5%)	5 (18%)	3 (10%)	5 (16%)	0 (0%)
Exhibitor space at conferences or events	22 (19%)	1 (5%)	10 (36%)	2 (7%)	8 (25%)	1(33%)

* Financial support includes sponsorship, for which no specific services are provided to the donor other than acknowledgement, and payment for services.

For peer review only

References:

1. Victora, C.G., Bahl, R., Barros, A.J., França, G.V., et al. Breastfeeding in the 21st century: epidemiology, mechanisms, and lifelong effect. *Lancet*, 2016; 387(10017), 475-490. https://doi.org/10.1016/S0140-6736(15)01024-7.

2. Rollins N.C., Bhandari N., Hajeebhoy N., Horton S., Lutter C.K., Martines J.C. et al (2106). Why invest, and what it will take to improve breastfeeding practices? *Lancet,* 387:491–504. doi:10.1016/S0140-6736(15)01044-2.

3. IMF (2017) World Economic Outlook http://www.imf.org/en/Publications/WEO/Issues/2017/04/04/world-economic-outlookapril-2017.

4. Save the Children Fund (2018). Don't push it: Why the formula milk industry must clean up its act. Retrieved from https://www.savethechildren.org.uk/content/dam/gb/reports/health/dont-push-it.pdf.

5. Taylor, A. (1998). Violations of the international code of marketing of breast milk substitutes: prevalence in four countries. *BMJ,* 316(7138): 1117–1122. Retrieved from https://www.ncbi.nlm.nih.gov/pmc/articles/PMC28512/.

6. Brady, JP (2012). Marketing breast milk substitutes: problems and perils throughout the world. *Archives of Disease in Childhood* 97:529-532. Retrieved from https://adc.bmj.com/content/97/6/529.

7. Shen, Y. (2016). Monitoring the International Code of Marketing of Breast-Milk Substitutes: A Comparative Analysis of Past Study Efforts. Retrieved from https://www.who.int/nutrition/events/2nd_comparative_analysis_studies_netcode_june2016.pdf?ua=1.

8. Secretaria de Salud, Estados Unidos Mexicanos; Subsecretaria de Prevención y Promoción de la Salud; Centro Nacional de Equidad de Género y Salud Reproductiva (2016). La Prevalencia de Violaciones al Código Internacional de Comercialización de Sucedáneos de la Leche Materna en México. Retrieved from https://www.gob.mx/cms/uploads/attachment/file/242297/ResumenViolacionesCICSLMorigEP.pdf.

9. Universidad de Chile, Departamento de Nutrición, Facultad de Medicina (2017). Monitoreo al Código Internacional de Comercialización de Sucedáneos de la Leche Materna en Santiago, Chile. Santiago, Chile. Retrieved from https://dipol.minsal.cl/wrdprss_minsal/wp-content/uploads/2018/02/INFORME-FINAL-MONITOREO-CICSLM-EN-CHILE-2017.pdf.

10. Fundación para el Bienestar y Atención Básica Social, Institucional y Comunitaria (FUNBBASIC), Ministerio de Salud Pública, UNICEF, PAHO/WHO (2017). Estudio para estimar la prevalencia de violaciones al Código Internacional de Comercialización de Sucedáneos de la Leche Materna en dos ciudades de Ecuador. Retrieved from

For peer review only

https://www.paho.org/ecu/index.php?option=com_docman&view=download&alias=695-estudio-violaciones-al-codigo-de-sucedaneos&category_slug=salud-familiar-y-comunitaria&Itemid=599.

11. U.S. Securities and Exchange Commission (2015). Press Release. SEC Charges Mead Johnson Nutrition with FCPA Violations. Retrieved from https://www.sec.gov/news/pressrelease/2015-154.html.

12. World Health Organization (WHO). (1981). *International Code of Marketing of Breast-milk Substitutes.* Retrieved from http://www.who.int/nutrition/publications/code_english.pdf.

13. World Health Organization, Fifty-Eighth World Health Assembly. (2005). Resolution WHA58.32 Infant and young child nutrition. Retrieved from http://www.who.int/nutrition/topics/WHA58.32_iycn_en.pdf?ua=1.

14. World Health Organization, Sixty-Ninth World Health Assembly. (2016a). Resolution WHA69.9 Ending inappropriate promotion of foods for infants and young children. Retrieved from http://apps.who.int/gb/ebwha/pdf_files/WHA69/A69_R9-en.pdf?ua=1&ua=1.

15. World Health Organization, Sixty-Ninth World Health Assembly. (2016b). Provisional agenda item 12.1 Guidance on ending the inappropriate promotion of foods for infants and young children. Retrieved from http://apps.who.int/gb/ebwha/pdf_files/WHA69/A69_7Add1-en.pdf?ua=1.

16. World Health Organization (WHO). (2017). Guidance on ending the inappropriate promotion of foods for infants and young children: Implementation manual. Retrieved from https://www.who.int/nutrition/publications/infantfeeding/manual-ending-inappropriate-promotion-food/en/.

17. International Paediatric Association. (2017). Member Societies. Retrieved from http://ipa-world.org/society.php.

18. Baby Milk Action: IBFAN UK. (2017). Infant formula: companies and brands. Retrieved from http://archive.babymilkaction.org/pages/milkcom.html.

19. World Health Organization (WHO). (2016c). WHO Framework of Engagement with Non-State Actors (FENSA). Retrieved from http://www.who.int/about/collaborations/non-state-actors/en/.

20. Rodwin, M.A., *Conflicts of Interest and the Future of Medicine: The United States, France and Japan*, Oxford: Oxford University Press, 2011. Pp. ix-375. ISBN 978 0 19 975548 6.

21. Fabbri, A., Gregoraci, G., Tedesco, D., Ferretti, F., Gilardi, F., Iemmi, D., Lisi, C., Lorusso, A., Natali, F., Shahi, E. & Rinaldi, A. (2016). Conflict of interest between professional medical societies and industry: a crosssectional study of Italian medical societies' websites. *BMJ Open, 6,* 1-8. Retrieved from http://bmjopen.bmj.com/content/bmjopen/6/6/e011124.full.pdf.

22. Costello, A., Branca, F., Rollins, N., Stahlhofer, M., Grummer-Strawn L. (2017). Health professional associations and industry funding. The Lancet, 389, 597-598. Retrieved from http://ac.els-cdn.com/S0140673617302775/1-s2.0-S0140673617302775-main.pdf?_tid=93dfdd3e-7076-11e7-a26a-00000aab0f26&acdnat=1500904204_1c57f80892d4e227e118453de636cc13.

For peer review only

23. Rothman, D., McDonald, W., Berkowitz, C., Chimonas, S., DeAgelis, C., Hale, R., Nissen, S., Osborn, J., Scully J., J., Thomson, G. & Wofsy, D. (2009). Professional Medical Associations and Their Relationships With Industry: A Proposal for Controlling Conflict of Interest. *Journal of American Medical Association, 301(13), 1367-1372.* Retrieved from http://jamanetwork.com.ezproxy2.library.colostate.edu/journals/jama/fullarticle/183670.

25. Van Tulleken C. (2018). Overdiagnosis and industry influence: how cow's milk protein allergy is extending the reach of infant formula manufacturers. *BMJ* 363 doi: https://doi.org/10.1136/bmj.k5056.

24. Dalsing, M. (2011). Industry working with physicians through professional medical associations. *Journal of Vascular Surgery, 54 (18S)*, 41S-46S. Retrieved from http://www.sciencedirect.com/science/article/pii/S0741521411013711?via%3Dihub.

26. Koletzko S., Niggemann B., Arato A., et al. (2012). Diagnostic approach and management of cow's-milk protein allergy in infants and children: ESPGHAN GI committee practical guidelines. Retrieved from http://spgp.pt/media/1066/pdf5-aplv.pdf.

27. Venter C., Brown T., Shah N., Walsh J., Fox A.T. (2013). Diagnosis and management of non-IgE-mediated cow's milk allergy in infancy - a UK primary care practical guide. *Clin Transl Allergy* 3:23.doi:10.1186/2045-7022-3-23 pmid:23835522.

28. Schofferman, J., Eskay-Auerbach, M., Sawyer, L., Herring, S., Arnold, P & Muehlbauer, E. (2013). Conflict of interest and professional medical associations: the North American Spine Society experience. *The Spine Journal, 13,* 974-979. Retrieved from http://www.sciencedirect.com.ezproxy2.library.colostate.edu/science/article/pii/S1529943012005530?via%3Dihub.

29. Sharfstein J.M. & Silver D.L. Relationships between the American Academy of Pediatrics and Infant Formula Companies. JAMA Pediatrics, 2017, E1-E2. Retrieved from http://jamanetwork.com/pdfaccess.ashx?url=/data/journals/peds/0/.

For peer review only

STROBE Statement—Checklist of items that should be included in reports of ***cross-sectional studies***

	Item No	Recommendation	Page #
**Title and abstract**	1	(*a*) Indicate the study's design with a commonly used term in the title or the abstract	3
		(*b*) Provide in the abstract an informative and balanced summary of what was done and what was found	3
**Introduction**			
Background/rationale	2	Explain the scientific background and rationale for the investigation being reported	4-5
Objectives	3	State specific objectives, including any prespecified hypotheses	5
**Methods**			
Study design	4	Present key elements of study design early in the paper	6-7
Setting	5	Describe the setting, locations, and relevant dates, including periods of recruitment, exposure, follow-up, and data collection	6
Participants	6	(*a*) Give the eligibility criteria, and the sources and methods of selection of participants	6
Variables	7	Clearly define all outcomes, exposures, predictors, potential confounders, and effect modifiers. Give diagnostic criteria, if applicable	7
Data sources/ measurement	8*	For each variable of interest, give sources of data and details of methods of assessment (measurement). Describe comparability of assessment methods if there is more than one group	7
Bias	9	Describe any efforts to address potential sources of bias	6
Study size	10	Explain how the study size was arrived at	6
Quantitative variables	11	Explain how quantitative variables were handled in the analyses. If applicable, describe which groupings were chosen and why	7
Statistical methods	12	(*a*) Describe all statistical methods, including those used to control for confounding	7
		(*b*) Describe any methods used to examine subgroups and interactions	7
		(*c*) Explain how missing data were addressed	7
		(*d*) If applicable, describe analytical methods taking account of sampling strategy	7
		(*e*) Describe any sensitivity analyses	N/A
**Results**			
Participants	13*	(a) Report numbers of individuals at each stage of study—eg numbers potentially eligible, examined for eligibility, confirmed eligible, included in the study, completing follow-up, and analysed	7
		(b) Give reasons for non-participation at each stage	N/A
		(c) Consider use of a flow diagram	N/A
Descriptive data	14*	(a) Give characteristics of study participants (eg demographic, clinical, social) and information on exposures and potential confounders	7
		(b) Indicate number of participants with missing data for each variable of interest	7
Outcome data	15*	Report numbers of outcome events or summary measures	7
Main results	16	(*a*) Give unadjusted estimates and, if applicable, confounder-adjusted estimates and their precision (eg, 95% confidence interval). Make clear	7-8

For peer review only - http://bmjopen.bmj.com/site/about/guidelines.xhtml

		which confounders were adjusted for and why they were included	
		(*b*) Report category boundaries when continuous variables were categorized	N/A
		(*c*) If relevant, consider translating estimates of relative risk into absolute risk for a meaningful time period	N/A
Other analyses	17	Report other analyses done—eg analyses of subgroups and interactions, and sensitivity analyses	N/A
**Discussion**			
Key results	18	Summarise key results with reference to study objectives	9
Limitations	19	Discuss limitations of the study, taking into account sources of potential bias or imprecision. Discuss both direction and magnitude of any potential bias	11
Interpretation	20	Give a cautious overall interpretation of results considering objectives, limitations, multiplicity of analyses, results from similar studies, and other relevant evidence	9-12
Generalisability	21	Discuss the generalisability (external validity) of the study results	11
**Other information**			
Funding	22	Give the source of funding and the role of the funders for the present study and, if applicable, for the original study on which the present article is based	2

*Give information separately for exposed and unexposed groups.

Note: An Explanation and Elaboration article discusses each checklist item and gives methodological background and published examples of transparent reporting. The STROBE checklist is best used in conjunction with this article (freely available on the Web sites of PLoS Medicine at http://www.plosmedicine.org/, Annals of Internal Medicine at http://www.annals.org/, and Epidemiology at http://www.epidem.com/). Information on the STROBE Initiative is available at www.strobe-statement.org.

For peer review only

For peer review only - http://bmjopen.bmj.com/site/about/guidelines.xhtml
